# Determinants of the access to remote specialised services provided by national sarcoma reference centres

**DOI:** 10.1186/s12885-021-08393-4

**Published:** 2021-05-29

**Authors:** Yohan Fayet, Raphaël Tétreau, Charles Honoré, Louis-Romée Le Nail, Cécile Dalban, François Gouin, Sylvain Causeret, Sophie Piperno-Neumann, Simone Mathoulin-Pelissier, Marie Karanian, Antoine Italiano, Loïc Chaigneau, Justine Gantzer, François Bertucci, Mickael Ropars, Esma Saada-Bouzid, Abel Cordoba, Jean-Christophe Ruzic, Sharmini Varatharajah, Françoise Ducimetière, Sylvie Chabaud, Pascale Dubray-Longeras, Fabrice Fiorenza, Sixtine De Percin, Céleste Lebbé, Pauline Soibinet, Paul Michelin, Maria Rios, Fadila Farsi, Nicolas Penel, Emmanuelle Bompas, Florence Duffaud, Christine Chevreau, Axel Le Cesne, Jean-Yves Blay, François Le Loarer, Isabelle Ray-Coquard

**Affiliations:** 1 Equipe EMS – Département de Sciences Humaines et Sociales, Centre Léon Bérard, F-69008 Lyon, France; 2 Univ Lyon, Université Claude Bernard Lyon 1, Université Saint-Étienne, HESPER EA 7425, F-69008 Lyon, F-42023 Saint-Etienne, France; 3 Medical Imaging Center, Institut du Cancer, Montpellier, France; 4 Department of Surgical Oncology, Institut Gustave Roussy, Villejuif, France; 5 Department of Orthopaedic Surgery, CHU de Tours, Faculte de médecine, Université de Tours, Tours, France; 6 Department of Clinical Research and Innovation, Centre Léon Bérard, Lyon, France; 7 Department of Surgery, Centre Léon Bérard, Lyon, France; 8 Department of Surgery, Centre Georges-Francois Leclerc, Dijon, Bourgogne France; 9 Department of Medical Oncology, Institut Curie, Paris, France; 10 Univ. Bordeaux, Inserm, Bordeaux Population Health Research Center, Epicene team, UMR 1219, F-33000 Bordeaux, France; 11 Clinical and Epidemiological Research Unit, INSERM CIC1401, Institut Bergonié, F-33000 Bordeaux, France; 12 Department of Pathology, Lyon University Hospital, Lyon, France; 13 Department of Medical Oncology, Institut Bergonié, 33000 Bordeaux, France; 14 Department of Medical Oncology, CHRU Jean Minjoz, Besançon, France; 15 Department of Medical Oncology, ICANS, Strasbourg, France; 16 Department of Medical Oncology, Institut Paoli-Calmettes, Marseille, France; 17 Orthopaedic and trauma department, Rennes1 University Pontchaillou University Hospital, Rennes, France; 18 Medical Oncology Department, University Côte d’Azur, Centre Antoine Lacassagne, Nice, France; 19 Radiation Oncology and Brachytherapy Department, Centre Oscar Lambret, Lille, France; 20 Department of Orthopaedic Surgery, Réunion University Hospital, St-Pierre, France; 21 Surgery Department, Centre François Baclesse, F-14000 Caen, France; 22 Equipe EMS, Centre Léon Bérard, F-69008 Lyon, France; 23 Oncology Department, Centre Jean Perrin, F-63011 Clermont-Ferrand, France; 24 Department of Orthopedics Traumatology, CHU de Dupuytren, F-87042 Limoges, France; 25 Medical Oncology Department, Hôpital Cochin; AP-HP, Cancer Research for PErsonalized Medicine (CARPEM); Paris University, Paris, France; 26 AP-HP Dermatology Department, Saint-Louis Hospital, INSERM U976, Université de Paris Diderot, Paris, France; 27 Department of Hepato-Gastroenterology and Digestive Oncology, Reims University Hospital, Reims, France; 28 Department of Radiology and Medical Imaging, CHU-hôpitaux de Rouen, Rouen, France; 29 Department of Medical Oncology, Cancer Institute of Lorraine, Alexis Vautrin, Vandoeuvre Les Nancy, France; 30 CRLCC Léon Berard – Lyon, Oncology Regional Network ONCO-AURA, Lyon, France; 31 Lille University Medical School and Centre Oscar Lambret, Lille, France; 32 Medical Oncology Department, ICO, Saint Herblain, Pays de la Loire France; 33 Department of Medical Oncology, CHU La Timone and Aix-Marseille Université (AMU), Marseille, France; 34 Department of Medical Oncology, ICR IUCT- Oncopole Toulouse, Toulouse, France; 35 Medical Oncology, Insitut Gustave Roussy, Villejuif, Ile-de-France France; 36 Departement of Medical Oncology, Centre Léon Bérard, Université de Lyon and Unicancer Paris, Lyon, France; 37 Department of Pathology, Institut Bergonié, Bordeaux, France; 38 Department of Medical Oncology, Centre Leon Berard, Lyon, Rhône-Alpes France

**Keywords:** Cancer inequalities, Spatial inequalities, Reference networks, Sarcoma, Cancer care accessibility, Rare cancers

## Abstract

**Background:**

Spatial inequalities in cancer management have been evidenced by studies reporting lower quality of care or/and lower survival for patients living in remote or socially deprived areas. NETSARC+ is a national reference network implemented to improve the outcome of sarcoma patients in France since 2010, providing remote access to specialized diagnosis and Multidisciplinary Tumour Board (MTB). The IGéAS research program aims to assess the potential of this innovative organization, with remote management of cancers including rare tumours, to go through geographical barriers usually impeding the optimal management of cancer patients.

**Methods:**

Using the nationwide NETSARC+ databases, the individual, clinical and geographical determinants of the access to sarcoma-specialized diagnosis and MTB were analysed. The IGéAS cohort (*n* = 20,590) includes all patients living in France with first sarcoma diagnosis between 2011 and 2014. Early access was defined as specialised review performed before 30 days of sampling and as first sarcoma MTB discussion performed before the first surgery.

**Results:**

Some clinical populations are at highest risk of initial management without access to sarcoma specialized services, such as patients with non-GIST visceral sarcoma for diagnosis [OR 1.96, 95% CI 1.78 to 2.15] and MTB discussion [OR 3.56, 95% CI 3.16 to 4.01]. Social deprivation of the municipality is not associated with early access on NETSARC+ remote services. The quintile of patients furthest away from reference centres have lower chances of early access to specialized diagnosis [OR 1.18, 95% CI 1.06 to 1.31] and MTB discussion [OR 1.24, 95% CI 1.10 to 1.40] but this influence of the distance is slight in comparison with clinical factors and previous studies on the access to cancer-specialized facilities.

**Conclusions:**

In the context of national organization driven by reference network, distance to reference centres slightly alters the early access to sarcoma specialized services and social deprivation has no impact on it. The reference networks’ organization, designed to improve the access to specialized services and the quality of cancer management, can be considered as an interesting device to reduce social and spatial inequalities in cancer management. The potential of this organization must be confirmed by further studies, including survival analysis.

## Introduction

Reference networks have been implemented in several European countries to improve the management of patients with rare cancers that require highly specialized diagnostic and therapeutic management to improve survival [1, 2]. According to the “hub-and-spoke” model, the reference networks’ organization is supposed to structure collaborations between a relatively high number of centres (spokes) ensuring geographical coverage and a limited number of reference centres (hubs) which concentrate the best expertise available, by “virtually centralizing some services (e.g. pathological diagnosis), referring some patients for selected procedures (e.g. surgery), directly carrying out other treatments (e.g. medical therapy), within a clinical strategy continuously shared with an Multidisciplinary Tumour Board (MTB)” [1, 3]. Sarcomas, which account for 1–3% of all cancers are paradigmatic models for rare cancers [4–6]. The complexity of these tumours requires a planned, coordinated and specialized initial management in order to ensure the best possible management and survival for these patients [7–10]. Reference networks organizing sarcoma management are currently operational in Scandinavian countries as well as in the United Kingdom [11, 12]. At the European scale, three European reference networks (ERN) dedicated to rare cancers have been launched in 2017: EuroBloodNet (https://www.eurobloodnet.eu), PaedCan (http://paedcan.ern-net.eu) and EURACAN (http://euracan.ern-net.eu). Each ERN brings together reference expert centres across Europe with a complete set of multidisciplinary expertise to facilitate the review of a patient’s diagnosis and treatment

Since the reference networks’ organization supports better access to expertise, it is important to assess and measure its potential beneficial effects on inequalities in cancer management. Previous studies showed worse survival for patients living in socially deprived and rural areas that can be related either to their lower rate of referral or to a later referral to specialized cancer centres [13–23]. Moreover, patients with rare cancer have worse survival than patients with common cancer and suffer from the lower accessibility of specialized facilities [24, 25]. By reducing the effects of barriers related to the patients’ place of residence, such as social deprivation and remoteness, which usually impede the early access to specialized services, the reference networks’ organization could therefore reduce inequalities in the cancer management.

In France, the sarcoma pathology (RRePS) and clinical (NetSarc) networks for visceral and soft tissue sarcomas were launched in 2010 and have been subsequently joined by RESOS focused on bone sarcomas. These three networks have since merged in a single NETSARC+ network, gathering together more than 30 reference centres. Following ESMO-EURACAN Clinical Practice Guidelines, each new sarcoma diagnosis should benefit from histological review and MTB discussion within a NETSARC+ centre during first-line management [26]. Remote access to these specialized services can be delivered thanks to the request of practitioners or facilities managing the patients.

Previous publications report the better compliance to international clinical guidelines, quality of initial management within the reference centres and its benefit on patients’ survival [7, 8, 27]. The IGéAS research program was designed to assess the ability of this national reference network to reduce geographical inequalities during the cancer management [25]. Using national sarcoma reference networks databases, the individual, medical and geographical factors associated with the early access to specialized services within the French sarcoma reference network NETSARC+ were analysed to determine whether sarcoma patient really benefit from this policy.

## Methods

### National sarcoma networks databases

All patients with specialized diagnosis and/or MTB discussion within a reference centre since 2010 are registered in a curated online national database approved by national health authorities (CNIL, n°910,390) (https://netsarc.sarcomabcb.org/). The databases contain 60 items divided into four themes: characteristics of the patient and tumour, diagnosis and review, key steps in management and follow-up, and successive presentations of the file and decision making at MTB. The municipality of patient, diagnosis and clinical data as well as patient follow-up are collected. A quality assurance program has been established for these databases to ensure the quality of medical data recorded, and clinical follow-up information is updated at least every 2 years.

### Constitution and analysis of the IGéAS cohort

The complete methodology of the IGéAS research program and description of the IGéAS cohort (*n* = 20,589) have been previously published [25]. The inclusion and exclusion criteria for the present work were as follow: patient living in France at time of diagnosis, diagnosis of sarcoma/GIST/desmoid tumour/intermediate malignancy tumour between the 1st of January 2011 and the 31th of December 2014. According to national sarcoma guidelines and the data collected in the NETSARC+ database, the steering committee of IGéAS research program has defined as follows:

1. Early access to specialized diagnosis as initial diagnosis or review performed in reference networks’ centres before 30 days of sampling
2. Early access to MTB discussion within NETSARC+ as first sarcoma MTB performed before the first surgery (open biopsy excluded). Radiation and/or chemotherapy used as neoadjuvant or even exclusive lines of therapy were considered as initial management.

Other patients who subsequently had access to review or MTB in the aftermath were recorded as late access.

### Statistical analysis

**Fig. 1:**
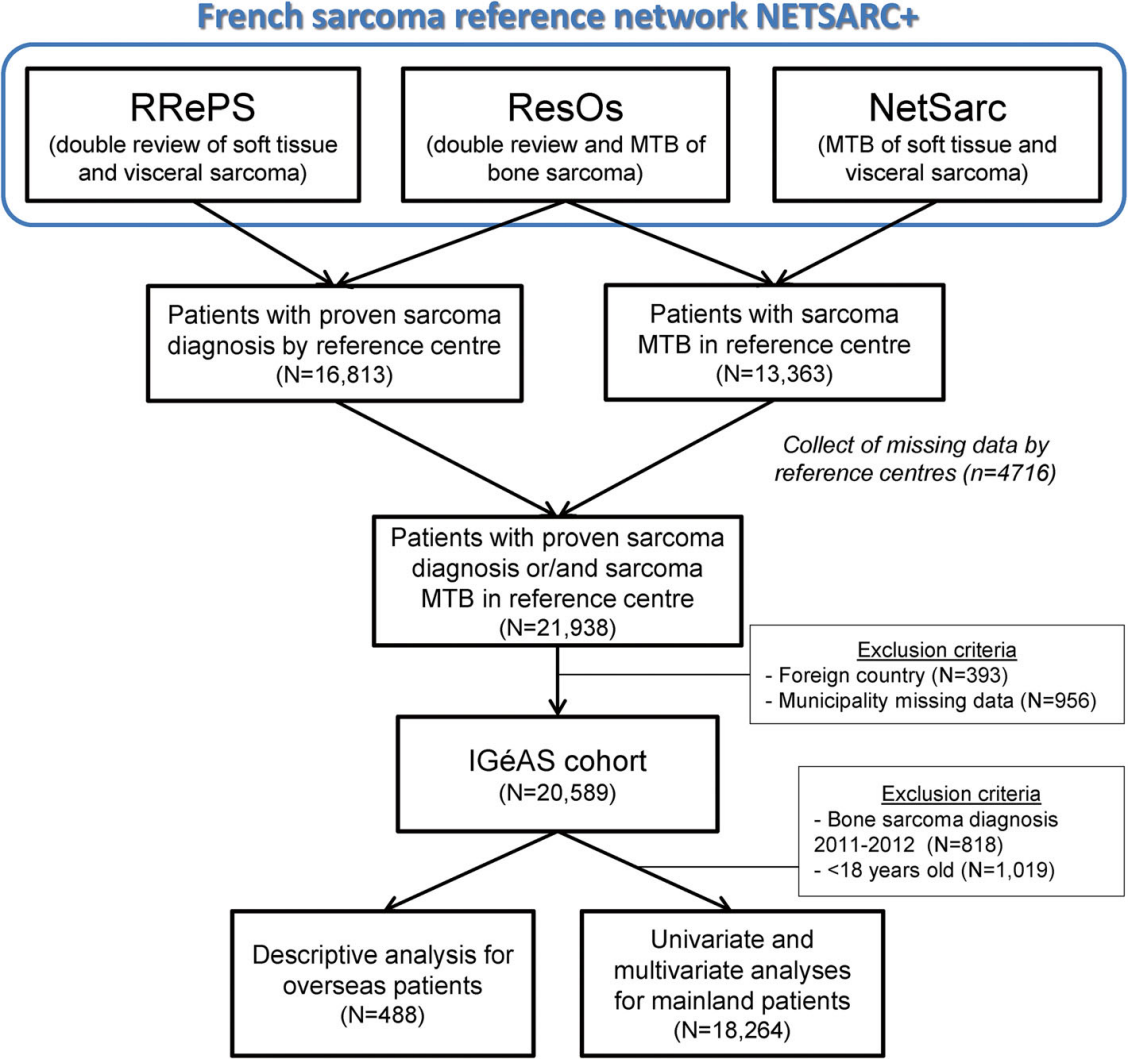
Flowchart of the constitution of the IGéAS cohort and data analyses

Univariate and multivariate analyses, following a binary logistic regression model, were performed to identify the factors associated with late access or no access to specialized diagnosis and clinical services. A total of 1837 patients (bone sarcoma diagnosis in 2011 and 2012 and patients under 18 years of age) of the IGéAS cohort were excluded from univariate and multivariate analyses because the corresponding populations was just partially recorded in the databases and might introduce some potential bias. Overseas patients were also excluded due to the lack of geographical data in overseas territories, limiting the calculation of geographical indices. As a result, univariate and multivariate analyses were done on 18,264 mainland patients. We provide descriptive analysis to assess the access to sarcoma specialized services in French overseas territories in comparison to mainland France (Fig. 1).

The univariate analyses used individual variables (sex, age) and clinico-pathological variables (past medical history, tumour size, pathological type, subtype and grade, depth of tumour, localization, year of diagnosis). For the “type of tumour” variable, we separated Gastro-Intestinal and Stromal Tumours (GIST) from other visceral sarcoma considering the specificities of GIST that have usually better prognosis. Using the patient’s municipality of residence at diagnosis, some validated geographic indices measuring the patients’ life context were also included: region, GeoClasH classification of the French municipalities [28], European Deprivation Index [29], population density [28], travel time to the closest reference pathological centre [25], travel time to the closest reference clinical centre, travel time to the closest general hospital [25] and localized potential accessibility index measuring spatial accessibility to general practitioners [30, 31]. Social information about the patients is not available in the NETSARC+ database to study the influence of social deprivation at the individual level.

All statistical analyses were performed using SAS software, version 9.4 (SAS Institute, Cary, USA). The candidate variables for the multivariate model were those with a *p*-value of less than 0.20 in univariate and with less than 20% missing data. Variables whose *p*-values were highlighted in grey were included in the multivariate analysis. All these variables are input into the model and then selected step by step (backward stepwise selection). The final model contains the variables that remain significant with a *p* < 0.05. Separated models with only clinical and geographical variables were finally performed to compare the respective impact of clinical and geographical variables on the access to the reference networks’ services. The adequacy and discrimination of the models were checked with the Akaiké Information Criterion (AIC), the percentage of well ranked and the area under the curve (0.5 indicates low discrimination and 1 indicates perfect discrimination).

## Results

### Comparison of the access to sarcoma specialized diagnosis and MTB between patients living in mainland France and overseas patients (IGéAS cohort)

A total of 11,642 of 20,101 (57.9%) and 199 of 488 (40.8%) sarcoma patients, respectively living in mainland France and the overseas territories, had access to a specialized diagnosis within Netsarc+ within 30 days of sampling (Table 1). A total of 6195 of 20,101 (30.8%) and 122 of 488 (25%) sarcoma patients, respectively from mainland France and overseas territories, had access to a specialized MTB within Netsarc+ before the first surgery.

**Table 1:** Access to sarcoma specialized services within Netsarc+ for French patients from 2011 to 2014 including overseas territories patients (source: IGéAS cohort, RRePS – ResOs – NETSARC databases)

	Access to specialized diagnosis			Access to specialized MTB		
	Early access	Late access	No access	Early access	Late access	No access
**Mainland France (*n*** **= 20,101)**	*N* = 11,642 (57.9%)	*N* = 4778 (23.8%)	*N* = 3681 (18.3%)	*N* = 6195 (30,8%)	*N* = 6905 (34,3%)	*N* = 7001 (34,8%)
**Overseas territories (*n*** **= 488)**	*N* = 199 (40.8%)	*N* = 193 (39.5%)	*N* = 96 (19.7%)	*N* = 122 (25,0%)	*N* = 140 (28,7%)	*N* = 226 (46,3%)

### Determinants of the initial management without access to sarcoma specialized diagnosis within Netsarc+

Sex, age, year of diagnosis, type, size, grade of tumour, geographic region and travel time to the closest reference centre for sarcoma diagnosis are associated with higher risk of initial management without access to sarcoma specialized diagnosis in Netsarc+ reference centres, in the final multivariate model (Table 2). Some clinical populations are at higher risk such as patients with non-GIST visceral sarcomas [OR 1.96, 95% CI 1.78 to 2.15] and patients with not graded sarcomas, according to the WHO classification of tumours [32] [OR 1.83, 95% CI 1.61 to 2.10]. We find no association with social deprivation and the farthest 20% of patients (more than 97 min of travel time to the closest reference centre for sarcoma diagnosis) have 18% higher risk of initial management without access to sarcoma specialized diagnosis [OR 1.18, 95% CI 1.06 to 1.31].

**Table 2:** Determinants of late access or no access to sarcoma specialized diagnosis within Netsarc+ from 2011 to 2014 (source: IGéAS cohort, RRePS – ResOs – NETSARC databases)

		Univariate (***N*** = 18,264)			Multivariate (***N*** = 18,264)		
Variables	late or no access / early access	OR	IC 95%	*p*-value	OR	IC 95%	*p*-value
**Individual and clinical variables**							
***Sex***				<.0001			0.0048
Male	3562/5656	1			1		
Female	3918/5128	1.21	[1.14;1.28]		1.09	[1.02;1.16]	
***Age***				<.0001			0.0249
> =75	1622/2711	1			1		
[18–25[	261/373	1.17	[0.98;1.38]		0.98	[0.82;1.18]	
[25–50[	1959/2536	1.29	[1.18;1.40]		1.12	[1.03;1.23]	
[50–75[	3638/5164	1.17	[1.09;1.26]		1.10	[1.02;1.19]	
***Year of diagnosis***				0.0018			0.0061
2011	1601/2315	1			1		
2012	1706/2501	0.98	[0.90;1.07]		0.95	[0.87;1.04]	
2013	1949/3017	0.93	[0.85;1.01]		0.9	[0.82;0.98]	
2014	2224/2951	1.09	[1.00;1.18]		1.03	[0.95;1.13]	
***Type of tumour***				<.0001			< 0.0001
Soft tissue	4666/7594	1			1		
Bone	474/578	1.33	[1.17;1.51]		1.10	[0.96;1.26]	
Viscera – GIST	917/1520	0.98	[0.89;1.07]		1.11	[0.99;1.26]	
Viscera - No GIST	1423/1092	2.12	[1.94;2.31]		1.96	[1.78;2.15]	
***Depth of tumour***				0,0007			
Superficial and deep	394/650	1					
Superficial	1526/2376	1.06	[0.92;1.22]				
Deep	4865/6873	1.16	[1.02;1.33]				
Missing	695/885	1.29	[1.10;1.52]				
***Size of tumour***				<.0001			< 0.0001
> =200	475/873	1			1		
[50–200[	3304/5008	1.21	[1.07;1.36]		1.15	[1.02;1.31]	
[0–50[	2236/3294	1.24	[1.10;1.41]		1.30	[1.14;1.48]	
Missing	1465/1609	1.67	[1.46;1.91]		1.51	[1.31;1.74]	
***Histotype category***				<.0001			
GIST	983/1621	1					
Sarcoma	4657/6179	1.24	[1.13;1.35]				
Tumour of intermediate malignancy	1840/2984	1.01	[0.92;1.12]				
***Grade***				<.0001			< 0.0001
1	749/1201	1			1		
2	2312/3784	0.98	[0.88;1.08]		0.89	[0.80;0.99]	
3	1266/1887	1.07	[0.95;1.20]		1.02	[0.91;1.15]	
Not applicable	1236/876	2.26	[1.99;2.56]		1.83	[1.61;2.10]	
Missing	1917/3036	1.01	[0.90;1.12]		0.93	[0.82;1.06]	
**Geographical variables**							
***Region***				<.0001			< 0.0001
Auvergne-Rhône-Alpes	1013/1319	1			1		
Nouvelle-Aquitaine	564/1527	0.48	[0.42;0.54]		0.46	[0.40;0.52]	
Pays-de-la-Loire	327/709	0.60	[0.51;0.70]		0.60	[0.51;0.71]	
Centre-Val de Loire	233/470	0.64	[0.54;0.77]		0.64	[0.54;0.77]	
Provence-Alpes-Côte-d’Azur	656/1241	0.68	[0.60;0.78]		0.69	[0.61;0.79]	
Bretagne	339/659	0.67	[0.57;0.78]		0.70	[0.59;0.82]	
Corse	46/76	0.78	[0.54;1.14]		0.72	[0.49;1.07]	
Occitanie	672/1131	0.77	[0.68;0.87]		0.78	[0.69;0.89]	
Bourgogne-Franche-Comté	333/538	0.80	[0.68;0.94]		0.83	[0.70;0.97]	
Grand-Est	732/838	1.13	[1.00;1.29]		1.18	[1.04;1.35]	
Hauts-de-France	737/744	1.29	[1.13;1.47]		1.31	[1.14;1.50]	
Normandie	301/287	1.36	[1.13;1.63]		1.43	[1.18;1.72]	
Ile-de-France	1527/1245	1.59	[1.42;1.78]		1.77	[1.57;2.00]	
***GeoClasH classification of municipalities***				<.0001			
Wealthy Metropolitan Areas	1824/2071	1					
Precarious Population Districts	3837/5748	0.75	[0.70;0.81]				
Residential Outskirts	913/1394	0.74	[0.67;0.82]				
Agricultural and Industrial Plains	582/968	0.68	[0.60;0.77]				
Rural Margins	324/603	0.61	[0.52;0.70]				
***Travel time to the closest reference centre for sarcoma diagnosis in minutes, quintiles)***				<.0001			0.0073
< = 21	1581/2119	1			1		
] 21; 47.5]	1548/2082	0.99	[0.90;1.09]		1.05	[0.95;1.16]	
] 47.5; 73.5]	1484/2187	0.90	[0.82;0.99]		1.10	[0.99;1.21]	
] 73.5; 97.5]	1491/2125	0.94	[0.85;1.03]		1.18	[1.06;1.31]	
> 97.5	1376/2271	0.81	[0.74;0.89]		1.18	[1.06;1.31]	
***European Deprivation Index (quintiles)***				< 0.0001			
< = − 1.3 (least deprived)	1478/2188	1					
]-1.3; 1.8]	1386/2227	0.92	[0.83;1.01]				
]1.8; 5.6]	1438/2248	0.94	[0.86;1.04]				
]5.6; 9.2]	1501/2218	1.00	[0.91;1.09]				
> 9.2 (most deprived)	1677/1903	1.30	[1.18;1.43]				
***Population density (number of inhabitants/km2, quintiles)***				< 0.0001			
< = 94.0926	1364/2304	1					
] 94.0926; 306.127]	1399/2226	0.68	[0.62;0.74]				
] 306.127; 1034.61]	1462/2184	0.72	[0.65;0.79]				
] 1034.61; 3693.94]	1547/2104	0.77	[0.70;0.84]				
> 3693.94	1708/1966	0.84	[0.77;0.92]				
***APL index (spatial accessibility to general practitioners, quintiles)***				< 0.0001			
< = 49.1 (lowest accessibility)	1528/2094	1					
] 49.1; 64]	1707/1973	1.18	[1.08;1.30]				
] 64; 78.4]	1408/2142	0.90	[0.82;0.99]				
] 78.4; 90.7]	1403/2345	0.82	[0.74;0.90]				
> 90.7 (highest accessibility)	1434/2230	0.88	[0.80;0.96]				

### Determinants of the initial management without access to sarcoma specialized MTB within Netsarc+

Age, year of diagnosis, type of tumour, depth, size of tumour, histotype category, grade, geographic region and travel time to the closest reference centre are associated with higher risk of late access or no access to sarcoma specialized MTB in Netsarc+ reference centres, in the final multivariate model (Table 3). The probability of optimal access to specialized MTB increased over time during the observation period (*p* < 0.0001). Some clinical populations are at higher risk such as patients with non-GIST visceral sarcoma [OR 3.56, 95% 3.16 to 4.01], with superficial [OR 2.15, 95 CI% 1.83 to 2.54] or less than 50 mm sized tumour s [OR 2.58, 95 CI% 2.23 to 2.98]. We found no association with social deprivation and the farthest 20% of patients (more than 102 min of travel time to the closest sarcoma reference centre) have 24% higher risk [OR 1.24, 95% CI 1.10 to 1.40] of initial management without access to sarcoma specialized MTB.

**Table 3:** Determinants of the late access (after first surgery) or no access to sarcoma specialized MTB within Netsarc+ from 2011 to 2014 (source: IGéAS cohort, RRePS – ResOs – NETSARC databases)

		Univariate (N = 18,264)			Multivariate (***N*** = 18,264)		
Variables	late or no access / early access	OR	IC 95%	*p*-value	OR	IC 95%	*p*-value
**Individual and clinical variables**							
***Sex***				0.0093			
Male	6421/2797	1					
Female	6460/2586	1.08	[1.02;1.16]				
***Age***				< 0.0001			< 0.0001
[18–25]	335/299	1			1		
[25–50]	2988/1507	1.77	[1.49;2.09]		1.33	[1.10;1.60]	
[50–75]	6286/2516	2.23	[1.89;2.62]		1.65	[1.38;1.99]	
> =75	3272/1061	2.75	[2.32;3.26]		1.85	[1.52;2.25]	
***Year of diagnosis***				< 0.0001			< 0.0001
2011	2972/944	1			1		
2012	3034/1173	0.82	[0.74;0.90]		0.78	[0.70;0.87]	
2013	3390/1576	0.68	[0.62;0.75]		0.79	[0.71;0.88]	
2014	3485/1690	0.65	[0.59;0.71]		0.68	[0.61;0.75]	
***Type of tumour***				< 0.0001			< 0.0001
Soft tissue	8187/4073	1			1		
Bone	415/637	0.32	[0.28;0.36]		0.35	[0.29;0.42]	
Viscera – GIST	2190/247	4.41	[3.84;5.05]		1.99	[1.32;3.00]	
Viscera - No GIST	2089/426	2.44	[2.18;2.72]		3.56	[3.16;4.01]	
***Depth of tumour***				< 0.0001			< 0.0001
Superficial and deep	667/377	1			1		
Superficial	3312/590	3.17	[2.721;3.7]		2.15	[1.83;2.54]	
Deep	7903/3835	1.16	[1.02;1.32]		0.82	[0.71;0.95]	
Missing	999/581	0.97	[0.82;1.14]		1.35	[1.09;1.66]	
***Size of tumour***				< 0.0001			< 0.0001
> =200	707/641	1			1		
[50–200]	5242/3070	1.54	[1.37;1.73]		1.40	[1.24;1.59]	
[0–50]	4486/1044	3.89	[3.43;4.42]		2.58	[2.23;2.98]	
Missing	2446/628	3.53	[3.07;4.05]		2.80	[2.39;3.28]	
***Histotype category***				< 0.0001			< 0.0001
Sarcoma	6809/4027	1			1		
GIST	2323/281	4.88	[4.29;5.56]		1.43	[0.96;2.14]	
Tumour of intermediate malignancy	3749/1075	2.06	[1.90;2.23]		2.07	[1.87;2.30]	
***Grade***				< 0.0001			< 0.0001
1	1204/746	1			1		
2	4373/1723	1.57	[1.41;1.75]		1.01	[0.90;1.10]	
3	1771/1382	0.79	[0.70;0.89]		0.92	[0.81;1.05]	
Not applicable	1304/808	1	[0.88;1.13]		0.94	[0.81;1.10]	
Missing	4229/724	3.61	[3.20;4.08]		2.59	[2.22;3.02]	
**Geographical variables**							
***Region***				< 0.0001			< 0.0001
Auvergne-Rhône-Alpes	1556/776	1			1		
Grand-Est	1051/519	1.01	[0.88;1.15]		0.91	[0.78;1.06]	
Nouvelle-Aquitaine	1457/634	1.14	[1.00;1.30]		0.93	[0.81;1.08]	
Bourgogne-Franche-Comté	594/277	1.06	[0.90;1.26]		0.94	[0.78;1.13]	
Centre-Val de Loire	484/219	1.10	[0.91;1.32]		0.98	[0.80;1.20]	
Pays-de-la-Loire	718/318	1.12	[0.96;1.31]		0.98	[0.82;1.17]	
Occitanie	1242/561	1.10	[0.96;1.26]		1.05	[0.91;1.21]	
Hauts-de-France	1024/457	1.11	[0.97;1.28]		1.08	[0.93;1.27]	
Ile-de-France	1991/781	1.27	[1.12;1.43]		1.12	[0.97;1.29]	
Bretagne	761/237	1.60	[1.35;1.89]		1.31	[1.09;1.59]	
Normandie	420/168	1.24	[1.02;1.52]		1.34	[1.08;1.67]	
Provence-Alpes-Côte-d’Azur	1485/412	1.79	[1.56;2.06]		1.52	[1.31;1.77]	
Corse	98/24	2.03	[1.29;3.20]		1.57	[0.95;2.58]	
***GeoClasH classification of municipalities***				0.1696			
Wealthy Metropolitan Areas	2763/1132	1					
Precarious Population Districts	6758/2827	0.97	[0.90;1.06]				
Residential Outskirts	1586/721	0.90	[0.80;1.00]				
Agricultural and Industrial Plains	1122/428	1.07	[0.94;1.22]				
Rural Margins	652/275	0.97	[0.83;1.13]				
***Travel time to the closest sarcoma reference centre (in minutes, quintiles)***				0.004			0.0013
< = 29	2576/1168	1			1		
[ 29; 56]	2555/1069	1.08	[0.98;1.19]		1.12	[1.00;1.25]	
[ 56; 79]	2561/1122	1.03	[0.93;1.14]		1.07	[0.96;1.20]	
[ 79; 102]	2507/1009	1.12	[1.01;1.24]		1.21	[1.08;1.36]	
> 102	2682/1015	1.19	[1.08;1.32]		1.24	[1.10;1.40]	
***European Deprivation Index (quintiles)***				0.04			
< = − 1.3 (least deprived)	2551/1115	1					
[-1.3; 1.8]	2527/1086	1.01	[0.92;1.12]				
[1.8; 5.6]	2648/1038	1.11	[1.00;1.23]				
[5.6; 9.2]	2584/1135	0.99	[0.90;1.09]				
> 9.2 (most deprived)	2571/1009	1.11	[1.00;1.23]				
***Population density (number of inhabitants/km2, quintiles)***				0.8876			
< = 94.0926	2579/1089	1					
[ 94.0926; 306.127]	2541/1084	0.99	[0.89;1.09]				
[ 306.127; 1034.61]	2566/1080	1.00	[0.90;1.10]				
[ 1034.61; 3693.94]	2597/1054	1.04	[0.94;1.15]				
> 3693.94	2598/1076	1.02	[0.92;1.12]				
***APL index (spatial accessibility to general practitioners, quintiles)***				0.8087			
< = 49.1 (lowest accessibility)	2546/1076	1					
[ 49.1; 64]	2575/1105	0.98	[0.89;1.08]				
[ 64; 78.4]	2517/1033	1.03	[0.93;1.14]				
[ 78.4; 90.7]	2666/1082	1.04	[0.94;1.15]				
> 90.7 (highest accessibility)	2577/1087	1.00	[0.90;1.10]				

### Respective impact of clinical and geographical variables on the access to reference networks’ services

Table 4 shows that models with only clinical (AIC = 24,149, 59.8% of well ranked observations, AUC = 0.59) or geographical variables (AIC = 24,116, 59.6% of well ranked observations, AUC = 0.60) have nearly the same quality to analyse the optimal access to specialized diagnosis in reference centres. These specific models are also less performative than the model with all (i.e. clinical and geographical) the variables (AIC = 23,258, 65.3% of well ranked observations, AUC = 0.65). Considering the access to specialized MTB, the quality of the model with only clinical variables (AIC = 18,910, 75.8% of well ranked observations, AUC = 0.75) is higher than the model with only geographical variables (AIC = 22,050, 53.9% of well ranked observations, AUC = 0.55) and is close to the model gathering all the variables (AIC = 18,852, 76.2% of well ranked observations, AUC = 0.76).

**Table 4:** Adequacy and discrimination parameters of the different logistic regression models (source: IGéAS cohort, RRePS – ResOs – NETSARC databases)

Models	Details	AIC	Well-ranked %	AUC
**Optimal access to diagnosis**	All variables	23,258	65.3	0.65
**Optimal access to diagnosis**	Clinical variables	24,149	59.8	0.59
**Optimal access to diagnosis**	Geographical variables	24,116	59.6	0.60
**Optimal access to MTB**	All variables	18,852	76.2	0.76
**Optimal access to MTB**	Clinical variables	18,910	75.8	0.75
**Optimal access to MTB**	Geographical variables	22,050	53.9	0.55

*AIC* (Akaike Information Criterion): The model to choose has the smallest AIC

Well-ranked %: The model to choose has the highest %

*AUC* (Area Under the Curve, from 0 to 1): The model to choose has the highest value

## Discussion

This study assessed the ability of the reference networks’ organizations, initially implemented to improve quality management and survival of rare cancers patients [8], to address in the same time some public health and social issues. Our aim was to provide a nationwide overview of the inequalities in the cancer management, in the specific setting of an accredited reference networks for rare cancers patients. A dedicated cohort was built for this study by cross-referencing databases recording pathological review and specialized MTB in reference centres to identify as many sarcoma patients as possible and find out under which conditions they were able to benefit or not from the expertise of the reference centres. Even if the databases of the French sarcoma reference networks support to reconsider upwards the incidence of sarcomas [5], only patients who have benefited from a pathological review or a discussion in sarcoma specialized MTB are recorded into the Netsarc+ databases. All incident sarcoma patients in France are therefore not included in this study, but we estimate the IGéAS cohort covers at least 90% of the national population [33]. Despite this limitation, our study is based on nationwide data gathering twenty thousand patients over 4 years and recording few dozens of individual and clinical information, which is quite original for rare cancers studies.

### The slight influence of social deprivation and distance to reference centres

In the context of national organization driven by reference network, distance to reference centres slightly alters the early access to sarcoma specialized services and social deprivation has no impact on it. This is an original finding with regards to the literature data on spatial inequalities in the cancer management [20, 21, 34–36]. For example, a nationwide study in the United States performed by Onega reported that “the most influential determinants of NCI-CC attendance were travel-time, place of residence, particularly for African Americans, and predominant type of care before diagnosis” rather than clinical factors included into the analysis like cancer site (breast, lung, colorectal or prostate cancer) or stage at diagnosis [36]. In the present study, the social deprivation of the municipalities has no impact on the early access to reference networks’ services. The use of deprivation indices at the IRIS (infra-municipality) scale or social information at the individual level would have supported a more accurate analysis of social inequalities but was not possible with available databases.

The distance to the nearest reference centre influences the access to specialized diagnosis and MTB but to a lesser extent in comparison with previous studies on the access to cancer-specialized facilities [20, 21, 36, 37]. Indeed, we found that patients living at more than 102 min to the closest reference centre have 18.3 and 24.4% higher risk of initial management without access to respectively sarcoma specialized diagnosis and MTB. As a comparison, Onega reported a decreased likelihood of 11% to attend NCI-Cancer Centre for every 10 min of added travel-time [OR 0.89, 95% CI 0.88 to 0.90] in the United States [36]. In France, where transportation costs can only be partially covered if the patient does not go to a local facility, Gentil showed that patients living more than 35 min away from the nearest reference care centre were 62% less likely [OR = 0.38, 95% CI 0.29; 0.50] to be operated on by a specialized surgeon than patients living less than 10 min away [21].

Regional inequalities in the early access to reference networks’ services must be cautiously interpreted because it could be related to heterogeneous practices in the databases’ recording depending on the reference centre, its intern organization and its own resources. It could also reflect the variable commitment of practitioners in this new structuring organization. Updating the regional inequalities on the basis of recent data would be relevant to determine whether this organization actually implies novel geographical inequalities at the regional scale, according to the variable adherence of local practitioners. Specific analysis and dedicated measures are needed to improve collaborations and networking between local facilities and reference centres in some regions as well as in the overseas territories, which suffer from the lack of reference centres on site.

Considering the specificities of sarcomas as well as the lower spatial accessibility of sarcoma reference centres in comparison to facilities usually managing cancers [25, 38], increased inequalities in the access to services may have been expected if reference networks were not implemented. Previous spatial analysis showed the large geographical coverage of the French sarcoma reference centres that are often requested to review specimens or to discuss therapeutic strategy of patients living several hundred kilometres away [25]. With regards to the literature, our results confirm the potential of reference networks to reach socially deprived and remote populations who usually suffer from the lower quality of their cancer management.

### Key insights to structure and improve the access to reference networks’ services

National recommendations of mandatory early pathology review and MTB discussion in a reference centre for all sarcoma diagnosis are not always complied with. Understanding and addressing the causes of this partial compliance with the national recommendations is a priority to improve the efficiency of the organization and the patients’ outcomes [39].

The overriding impact of the clinical factors on the access to sarcoma reference networks’ services suggests that first-line practitioners refer their patients to reference centres according to the clinical setting of their patients. According to guidelines, all new sarcoma diagnosis should benefit from a specialized pathological diagnosis as well as a specialized MTB within a NETSARC+ centre. First-line practitioners may probably consider that the early use of the reference networks’ services is not always necessary depending on their evaluation of the clinical situation of the patient and may be concerned that it will delay the management of sarcoma patients. This unframed practice of selection by non-specialist sarcoma practitioners led to an underuse of the reference networks’ services and can have serious effects in the management of patients.

Moreover, dedicated actions should target specific populations that suffer from an insufficient access to sarcoma expertise. For example, patients with non-GIST visceral sarcoma have much higher risk of late or no access to sarcoma specialized diagnosis and MTB within NETSARC+, while these sarcomas are particularly aggressive (only 55% 3-year survival rate in the IGéAS cohort). This finding could be related to the management of cancer based on their anatomical location or management through “organ-specific” health care management organization, which refer only secondarily patients to the sarcoma network after changes of histological diagnosis.

## Conclusion

In the context of national organization driven by reference network, geographical characteristics (social deprivation, remoteness) usually impeding the optimal management of cancers patients have much lower impact on the access to specialized services. While many countries are struggling to address cancer inequalities, the potential of the reference networks’ organization to reduce of inequalities in the cancer management must be confirmed by further studies, including survival analysis.

## Data Availability

The data that support the findings of this study are available from the French Sarcoma Reference Network NETSARC+ but restrictions apply to the availability of these data, which were used under license for the current study, and so are not publicly available. Data are however available from the authors upon reasonable request and with permission of the French Sarcoma Reference Network NETSARC+.
